# What Should Be Learned From Repurposed Antivirals Against SARS-CoV-2?

**DOI:** 10.3389/fmicb.2022.843587

**Published:** 2022-02-16

**Authors:** Miguel Angel Martinez

**Affiliations:** IrsiCaixa, Hospital Universitari Germans Trias i Pujol, Universitat Autònoma de Barcelona, Badalona, Spain

**Keywords:** antiviral, repurposed drugs, virus therapy, SARS-CoV-2, emerging viruses

Since severe acute respiratory syndrome coronavirus 2 (SARS-CoV-2) was first identified in 2019, more than 270 million infections have been reported, and the death toll from the associated coronavirus disease 2019 (COVID-19) has reached 5.5 million. The magnitude of this pandemic has encouraged the repurposing of numerous drugs, with the aim of rapidly curbing the morbidity, mortality, and spread of this new disease. However, this approach has had limited success. Only vaccine development and the repurposing of dexamethasone have impacted COVID-19 severity. Drug repurposing is, perhaps, an effective strategy for identifying antivirals, but there are no shortcuts for drug development. The identification of effective drugs, regardless of previous approval, requires time and funding to confirm and understand the drug target, the drug's toxicity, and its proper use. The lessons learned with COVID-19 should provide a roadmap for approaching drug discovery, when confronting SARS-CoV-2 and other current and future viral threats.

From the beginning of the SARS-CoV-2 pandemic in China, it was clear that severe COVID-19 was an inflammatory acute respiratory distress syndrome (ARDS). Indeed, early in the pandemic, COVID-19 progression from mild to moderate to severe was associated with immune dysfunction, hyper-inflammatory response, and sepsis (Zhou et al., 2020). Later studies showed that some patients infected with SARS-CoV-2 developed severe pneumonia and ARDS (Grant et al., 2021). Consequently, both antiviral and immunomodulatory drugs were contemplated and administered early in the pandemic (Martinez, 2020; Cross et al., 2021). Early studies (Tay et al., 2020) searched for parallelisms between SARS-CoV-2-related pneumonia and pneumonias connected to other human coronavirus diseases, such as severe acute respiratory syndrome (SARS-CoV) and Middle East respiratory syndrome (MERS-CoV). However, the pathobiology of SARS-CoV-2-related pneumonia seems to be distinct from pneumonia caused by other respiratory viral and bacterial pathogens; the SARS-CoV-2-related pneumonia displays unusual clinical features and has a longer clinical course than severe pneumonia (Gattinoni et al., 2020; Grant et al., 2021). An accurate clinical characterization of SARS-CoV-2 infections, based on known clinical syndromes, is critical for the identification of effective therapies (Cross et al., 2021).

Understandably, early in the SARS-CoV-2 pandemic, several repurposed drugs were quickly translated to the clinic. However, over the 2 last years, the results have been rather poor (Edwards, 2020; Edwards and Hartung, 2021; Martinez, 2021). The repurposed drugs for combating COVID-19 were selected either without a hypothetical basis or with mechanistic hypothetical approaches. Unfortunately, when those candidates were tested in clinical studies, none proved effective against SARS-CoV-2. Given that the repurposed drugs were optimized for a different target, dosage, or tissue, their pharmacology was probably not appropriate for the new indication (Edwards, 2020; Dahlin et al., 2021). In addition, the cellular assays designed for repurposing antivirals, including phenotypic and high-throughput screening assays, are frequently subject to interfering or undesirable bioactive mechanisms (Dahlin et al., 2021). Remarkably, most of the repurposed drugs tested during this pandemic were administered off-label and outside of properly designed observational or clinical trials. However, unsuccessful approaches might reduce the public trust in evidence-based medicine (Cross et al., 2021). Therefore, clinical trials and methodologies for identifying effective drugs should be designed based on methodologies with the highest likelihood of success.

Antiviral drugs target key processes in the virus life cycle. These processes include virus cell entry, genome replication, translation, protein processing, and virus particle generation. More than two-thirds of all antivirals approved for human use target viral enzymes, such as viral RNA/DNA polymerases and viral proteases (De Clercq and Li, 2016; Tompa et al., 2021). Importantly, in the last 30 years, no antiviral drug has been approved in the absence of a specific viral target. Thus, perhaps the main drawback of the repurposed antiviral drugs that have been tested unsuccessfully against SARS-CoV-2 has been the absence of an evident mechanism of action (Table 1).

**Table 1:** Most relevant repurposed antiviral drugs against SARS-CoV-2.

**Antiviral drug**	**Approved target disease**	**Drug target**	**COVID-19 clinical trial reference**
Favipiravir	Influenza virus	Virus RdRp	Doi et al., 2020
Hydroxychloroquine	Malaria	Hemozoin biocrystallization	Axfors et al., 2021; WHO Solidarity Trial Consortium, 2021
Ivermectin	Antiparasitic	Glutamate-gated chloride channels	Baker and Reardon, 2021; Popp et al., 2021
Interferon beta-1a	Multiple sclerosis	Reduce inflammation	WHO Solidarity Trial Consortium, 2021
Lopinavir	HIV-1	Virus protease	Cao et al., 2020; WHO Solidarity Trial Consortium, 2021
Remdesivir	SARS-CoV-2	Virus RdRp	Wang et al., 2020; WHO Solidarity Trial Consortium, 2021

Hits from antiviral screening based on biochemical or cell-culture assays must be treated with skepticism. Chloroquine and hydroxychloroquine represent a clear example of misinterpreted cell culture results. The efficacy of chloroquine and hydroxychloroquine against SARS-CoV-2 in tissue cultures was determined with African green monkey kidney-derived Vero cells. However, when Vero cells were engineered to express TMPRSS2, a cellular protease that activates SARS-CoV-2 for entry into lung cells, the genetic manipulation rendered SARS-CoV-2–infected Vero cells insensitive to chloroquine (Hoffmann et al., 2020). Accordingly, chloroquine could not block SARS-CoV-2 infections in TMPRSS2-expressing human lung Calu-3 cells; thus, chloroquine targets a viral activation pathway that is not present in human lung cells. Therefore, the drug is unlikely to protect against the spread of SARS-CoV-2. Another example of a misinterpretation of cell culture results was recently reported (Tummino et al., 2021) in a study that demonstrated that phospholipidosis was a shared mechanism underlying the antiviral activity of many repurposed drugs for SARS-CoV-2 (Tummino et al., 2021). Conversely, drugs that were active against the same targets, but did not induce phospholipidosis, were not effective against viruses. Those results strongly suggested that a failure to induce phospholipidosis could explain why most drugs that were selected for repurposing to date lacked clinical efficacy against SARS-CoV-2. In future pandemics, the target specificity of candidate antivirals should be examined before initiating treatment to exclude non-specific mechanisms during drug development. Target specificity should be accompanied by safety profiles and a determination of the pharmacokinetics/pharmacodynamics of selected drugs. Viruses manipulate many cellular processes during their life-cycles; thus, when a tested drug modifies a cellular pathway, it frequently leads to a false-positive hit.

Even when a drug mechanism of action is known, the preclinical data must be interpreted with caution. At the beginning of the SARS-CoV-2 pandemic, remdesivir, developed by Gilead, was the most promising antiviral drug for combating SARS-CoV-2. Initial interest in the drug was based on its potency in cell culture models of SARS-CoV-2, including primary human airway epithelial cells (Pruijssers et al., 2020). Moreover, remdesivir displayed specificity against the RNA-dependent RNA polymerase (RdRp) of other coronaviruses (Agostini et al., 2018), such as SARS-CoV and MERS-CoV. Moreover, it showed prophylactic and therapeutic efficacy in a rhesus macaque model of a MERS-CoV infection (de Wit et al., 2020). However, the clinical efficacy of remdesivir for COVID-19 remains controversial. Several trials have found no significant differences between remdesivir-treated and control groups in the time-to-clinical-improvement or mortality (Yan and Muller, 2021). Previous work with remdesivir also showed impressive preclinical and animal-model results against Ebola virus (Warren et al., 2016); however, those results were not confirmed in clinical studies (Mulangu et al., 2019).

In severe cases, SARS-CoV-2 induces an overexpression of inflammatory cytokines. Accordingly, immunomodulatory therapies, such as Anakinra (anti-interleukin 1b) and Tocilizumab (anti-interleukin 6), were repurposed for COVID-19. However, monotherapies with these compounds were unsuccessful. Remarkably, broad-spectrum immune-modulators, such as dexamethasone or baricitinib (anti-Janus kinase) have shown better efficacy in severe COVID-19 cases (Tomazini et al., 2020; Abani et al., 2021; Kalil et al., 2021). The pathogenesis of COVID-19 is different from those of other respiratory diseases. Consequently, repurposing immunomodulatory therapies might be more difficult than initially thought. Additionally, immunomodulatory therapies that might be useful for severe COVID-19 might be harmful in mild or moderate COVID-19, where the immune system can work effectively to control viral replication and disease (Cross et al., 2021).

Historically, repurposing drugs has only been successful in a few cases (Edwards, 2020). Examples are aspirin, for treating coronary artery disease; erythromycin, for treating impaired gastric motility; sildenafil for treating erectile dysfunction; thalidomide for treating multiple myeloma; and imatinib for treating gastrointestinal stromal tumors (Corsello et al., 2017). Most of those achievements were serendipitous. Nevertheless, those chance discoveries have promoted systematic searches for other drugs that might be repurposed. These explorations have mainly relied on high-throughput screening technologies and computational modeling with large datasets (Edwards, 2020). Currently, several libraries of approved drugs are available for rapid screening to identify candidates for repurposing against the targeted disease. Unfortunately, systematic, hypothesis-free, large-scale screening of these drug libraries has yet to yield effective treatments for most targeted diseases.

Effective vaccines against SARS-CoV-2 have been developed with extraordinary speediness. Vaccines have prevented COVID-19 development, but they have not generated sterilizing immunity in a significant percentage of individuals; thus, viral transmission remains possible after a vaccination (Krammer, 2020; Cromer et al., 2021). The continuous emergence of SARS-CoV-2 variants, such as the Delta or Omicron variant, has challenged the efficacy of current vaccines. The Omicron outbreak in southern Africa might be due to its capacity to infect people that have been vaccinated or have recovered from COVID-19 caused by Delta and other variants. At this stage, there is a need for an antiviral that effectively combats SARS-CoV-2 to prevent more severe disease, hospitalizations, and deaths.

A combination of vaccines and antiviral drugs should be a powerful tool for controlling the morbidity, mortality, and spread of SARS-CoV-2. Antivirals should target SARS-CoV-2 infections early to prevent rapid viral replication. In addition to the approved antiviral, remdesivir, which is delivered intravenously, two additional oral drugs, molnupiravir and nirmatrelvir (paxlovid), have completed human phase 3 clinical trials and have been approved (Table 2). Molnupiravir, a cytidine ribonucleoside analog first developed to inhibit influenza virus replication, was shown to inhibit the SARS-CoV-2 RdRp (Painter et al., 2021). Molnupiravir can pair ambiguously, as cytidine or uridine, with viral RNA. This pairing introduces an elevated mutation load that generates non-infectious virus particles. Molnupiravir, promoted by Merck for SARS-CoV-2 clinics, has the potential to be a broad-based inhibitor; it acts against a number of RNA viruses, including seasonal influenza virus, MERS-CoV, and encephalitic alphaviruses, such as Venezuelan, Eastern, and Western equine encephalitis viruses. Phase 3 clinical trial data from Merck have shown that a 5-day course of molnupiravir reduced hospitalization or mortality by ~50%, compared to placebo, in patients with mild or moderate COVID-19. Nevertheless, the efficacy of molnupiravir was reduced from 50 to 30% in the published peer-reviewed data of the former phase 3 trial (Jayk Bernal et al., 2021). Nirmatrelvir inhibits the main SARS-CoV-2 protease (Owen et al., 2021). Protease inhibitors have been shown to be powerful antivirals, as demonstrated with human immunodeficiency virus type 1 (HIV-1) and hepatitis C virus (HCV). In a phase 3 clinical trial, nirmatrelvir was combined with ritonavir, an HIV-1 protease inhibitor that prevents enzymes in the liver from breaking down the antiviral. This combination showed 89% efficacy in preventing COVID-19 hospitalization and death. Still, the data for nirmatrelvir, generated by Pfizer, have not yet been peer-reviewed. These two promising antivirals will probably be effective against SARS-CoV-2 variants of concern, because these variants are characterized by mutations in the viral spike protein and other regions that are targeted by the immune system and/or by vaccines. Nevertheless, it should not be overlooked that monotherapy with any of these antivirals may induce viral drug resistance. This has been a familiar problem with other virus infections, like HIV-1 and HCV, which require treatments with a combination of antiviral drugs.

**Table 2:** Approved direct acting antiviral drugs against SARS-CoV-2.

**Antiviral drug**	**Drug target**	**Delivery**	**References**
Molnupiravir (MK-4482/EIDD-2801)	Virus RdRp	Oral	Jayk Bernal et al., 2021
Nirmatrelvir (PF-07321332)	Virus main protease	Oral	Owen et al., 2021
Remdesivir (GS-5734)	Virus RdRp	Intravenous	Wang et al., 2020; WHO Solidarity Trial Consortium, 2021

The emergence of new human viruses, such as HIV-1, HCV, Ebola virus, and Zika virus, has taught us that every new emerging virus has a distinct life style and a pathogenic course that was unknown in preceding viral infections. Moreover, SARS-CoV-2 is likely to become a permanent, endemic virus, like the previous, recently emerged human viruses. Repurposing drugs has been an appealing strategy for the rapid translation of drugs to the clinic. However, despite the enthusiasm for this strategy, it has not produced any effective treatments for COVID-19, or any other viral disease, including those caused by recent emergent viruses, such as Zika virus and Ebola virus. Drug development requires time, funding, and collaboration among clinical investigators, virologists, immunologists, and pharmacologists. It is important to know and understand the drug's target, toxicity, and pharmacodynamics, in addition to the key viral kinetic time-points and specific (or broad) immune phenomena. This approach may take more time, but it could be useful for fighting SARS-CoV-2 and for developing drugs to combat newly emerging viruses and future pandemics.

## Author Contributions

The author confirms being the sole contributor of this work and has approved it for publication.

## Funding

This work was supported by the Spanish Ministry of Science and Innovation (PID2019-103955RB-100).

## Conflict of Interest

The author declares that the research was conducted in the absence of any commercial or financial relationships that could be construed as a potential conflict of interest.

## Publisher's Note

All claims expressed in this article are solely those of the authors and do not necessarily represent those of their affiliated organizations, or those of the publisher, the editors and the reviewers. Any product that may be evaluated in this article, or claim that may be made by its manufacturer, is not guaranteed or endorsed by the publisher.
